# Changes in Odor Background Affect the Locomotory Response to Pheromone in Moths

**DOI:** 10.1371/journal.pone.0052897

**Published:** 2013-01-02

**Authors:** Virginie Party, Christophe Hanot, Daniela Schmidt Büsser, Didier Rochat, Michel Renou

**Affiliations:** UMR 1272 Physiologie de l’Insecte Signalisation et Communication, INRA, Versailles, France; Barnard College, Columbia University, United States of America

## Abstract

Many animals rely on chemical cues to recognize and locate a resource, and they must extract the relevant information from a complex and changing odor environment. For example, in moths, finding a mate is mediated by a sex pheromone, which is detected in a rich environment of volatile plant compounds. Here, we investigated the effects of a volatile plant background on the walking response of male *Spodoptera littoralis* to the female pheromone. Males were stimulated by combining pheromone with one of three plant compounds, and their walking paths were recorded with a locomotion compensator and analyzed. We found that the addition of certain volatile plant compounds disturbed the orientation toward the sex pheromone. The effect on locomotion was correlated with the capacity of the plant compound to antagonize pheromone detection by olfactory receptor neurons, suggesting a masking effect of the background over the pheromone signal. Moths were more sensitive to changes in background compared to a constant background, suggesting that a background odor also acts as a distracting stimulus. Our experiments show that the effects of odorant background on insect responses to chemical signals are complex and cannot be explained by a single mechanism.

## Introduction

Insects rely largely on chemical cues to recognize and locate essential resources. Their olfactory system must extract the relevant information from a complex and changing odor environment. For instance, in moths, mate finding is mediated by a volatile pheromone released by the female in minute amounts. This sex pheromone is a blend of a few components whose chemical structure and ratios ensure species-specificity. Its perception triggers highly predictable behaviors in males [1], a general activation that is followed by take-off, oriented flight, landing close to the source, close-range approach by walking, and finally courtship and mating attempts [2], [3].

The plants that constitute a significant part of the natural environment of moths emit a great diversity of volatile compounds (PV) in large amounts. PV emissions vary from plant species to plant species, and for the same individual plant, they fluctuate according to the physiological state [4] or the circadian rhythm [5], [6]. A number of these PVs have a signaling value for phytophagous insects. Host plant PVs provide cues for food sources or oviposition sites [7]. Some other PVs, emitted by non-host plants or host plants under herbivore attack repel insects [8]. With the varying amount of volatiles produced by individual plants and a highly variable combination of different plants in their natural habitats, the olfactory environment of a moth is complex and continuously changing. It creates an unpredictable odorant background that can interact in different ways with the perception of specific signals [9]. Thus, analyzing the effects of mixtures of pheromones and PVs constitute a unique opportunity to unravel how the olfactory system operates to extract relevant information from an apparently blurry chemical world, leading to the appropriate behavior.

In addition to their important role in foraging and oviposition behaviors, PVs have long been acknowledged to modulate insect pheromone communication [10], [11]. Laboratory and field experiments have shown that PVs may enhance or decrease the attraction of male moths to pheromone sources when mixed with the pheromone. Synergy between pheromone and plant compounds identified from the volatile emission of larval host plants has been observed in *Spodoptera exigua* [12], *Helicoverpa zea, Cydia pomonella* [13] and *Eupoecilia ambiguella* [14]. On the contrary, the inhibition of attraction by non-host volatiles has been reported by Jactel and coworkers [15] in *Thaumetopoea pityocampa*. Branches of birch, a non-host tree, or diffusers of methyl salicylate, a major compound of birch effluvia, significantly reduced the number of males caught in pheromone traps when deposited at the base of the trunk of the host tree that supported the trap.

Pheromones and PVs have long been believed to be detected via separated receptor neurons and the resulting sensory input transmitted via labelled lines to different structures in the primary olfactory centers, the antennal lobes (ALs) [16]. Behavioral effects due to mixture interactions have therefore been thought to occur mainly through the integration of PVs and pheromones in higher centers within the brain. The literature shows, however, that interactions between pheromones and PVs already take place at the level of olfactory receptor cells. For example, linalool and Z(3)-hexenyl acetate were shown to synergize the response of olfactory receptor neurons to the main pheromone compound in male *Helicoverpa zea* [17]. However, several other studies have reported a reduction of the firing response to pheromones in the presence of various compounds of plant origin [18].

Interestingly, PVs do not necessarily have to be presented simultaneously with the pheromone to affect behavior. A brief and single exposure to linalool 24 h before pheromone presentation increases the attraction to the natural pheromone of male *Spodoptera littoralis* walking on a locomotion compensator [19]. The authors concluded that the enhanced response was due to general sensitization, regardless of the modality of the stimulus. This result suggests that behaviorally relevant stimuli in the environment contribute to a maturation process in the moth sensory systems. Thus, the plant odorant context in which insects live is not only important for their immediate responses, but it may durably affect their sensitivity to sensory signals.

While it is now well acknowledged that host PVs affect pheromone communication in male moths, their possible modes of action are still obscure and most likely multiple. We previously showed that backgrounds of linalool and some other PVs reduce the responses of olfactory receptor neurons tuned to pheromone components (Ph-ORNs) [18], while preserving the temporal coding of pheromone pulses [20] in male *S. littoralis.* Furthermore, the introduction of linalool in the background resulted in changes in the speed and direction of male *S. littoralis* walking toward a pheromone source [20]. This prompted us to more precisely analyze the disturbing effects of an odor background on the pheromone-triggered walking response (PWR). As working hypotheses, we postulate that background odors change pheromone-mediated walking paths in male moths as a result of two different, although not mutually exclusive, mechanisms: masking and distraction. The masking hypothesis puts the emphasis on the peripheral effects of the background. Because a strong general odorant background interferes negatively with the detection of the pheromone, jamming the olfactory system, it reduces the perceived intensity of the pheromone signal. Thus, each change in the background should significantly impact the PWR. The distraction hypothesis deems that the perception of an odorant background may reallocate some of the animal’s finite attention to its olfactory environment, acting as a distracting stimulus [21], [22], [23] for the male moth engaged in oriented locomotion toward the pheromone source. We then expected that the effects of background changes on the response would decrease with the repetition of the distracting stimulus.

To test the two hypotheses, we designed experiments in which we analyzed the PWR of male *S. littoralis* recorded on a locomotion compensator during stimulation. An odor background was either applied simultaneously with the pheromone (one phase experiments), one odorant was added to another, or one odorant was removed from the mixture during orientation (two- and multi-phase experiments). After detecting the pheromone in natural conditions, males first fly upwind, then land close to the source and approach by walking. We chose to analyze this walking response with a locomotion compensator because the odor stimulus can be precisely focused on the walking insect during the full time course of the experiment, and the locomotion can be recorded over an unlimited distance and duration. Thus, this device guarantees a satisfactory control of the exposure of the insect to both odors and allows several fast changes of stimuli. The suitability of the locomotion compensator to quantify moth locomotor responses to pheromone has been previously demonstrated in three moth species, *Bombyx mori* [3], *Manduca sexta* [2] and *S. littoralis* [19], [20], which actively walked towards pheromone sources. In the noctuid moth *S. littoralis*, this set-up has proven to be efficient in revealing changes in responsiveness after pre-exposure [19] and loss of orientation in response to the application of linalool [20].

Three PVs were chosen as representative molecules for different chemical structures and distinct ecological significance to *S. littoralis*. Linalool is a monoterpene, constitutively present in the volatile emissions of host plants and flowers visited by *S. littoralis* [24]. *Cis*-3-hexenyl acetate is a short-chain fatty acid derivative, contributing to the “grassy” odor, and it is emitted by numerous host plants of *S. littoralis,* including cotton [25]. Isoprene is abundantly released by deciduous trees [26] and it contributes to the natural odorant background in which *S. littoralis* lives, but its broad distribution makes it a poor cue for a specific resource for the moth.

We also recorded the firing activity of pheromone-sensitive olfactory receptor neurons in response to the *S. littoralis* sex pheromone in neutral or odorized backgrounds to compare the masking activity of the three PVs.

## Materials and Methods

### Insects

*Spodoptera littoralis* were reared in the laboratory on an artificial diet at 22°C, 60 to 70% relative humidity and under a L16:D8 photoperiod until emergence. Sexes were separated at the pupal stage and maintained in different climate-controlled chambers under a reverse LD regime. Newly emerged adults were collected every morning. Male adults were provided with a 10% sucrose solution. One- or 2-day-old males were used for electrophysiological studies, and 2-day-old males were used for behavioral studies.

### Stimulus Chemicals

(*Z*,*E*)-9,11-tetradecadienyl acetate (“Phero”; >97% purity checked by gas chromatography, CAS 50767-79-8) was synthesized in the laboratory (courtesy of Martine Lettere). Dilutions were prepared in hexane (>98% purity, CAS 110-54-3) from Carlo-Erba (Val-de-Reuil, France).

Linalool (“Lin”; racemic, 97% purity, CAS 78-70-6) and isoprene (“Iso”; ≥98% Purity, CAS 78-79-5), were purchased from Fluka Analytical and Merck, respectively, (Sigma-Aldrich, L’Isle-d’Abeau, France). *Cis*-3-Hexenyl Acetate (“Hex:Ac”; 99% purity, CAS 3681-71-8) was purchased from Lancaster Synthesis (Alpha Caesar, USA).

White mineral oil from Sigma (“MO”; CAS 8042-47-5) was used to prepare volume-to-volume dilutions of the PVs.

For preparing extracts of the natural pheromone, 20 to 30 glands from 2-day-old virgin females were dissected and extracted in 100 µL hexane 2 to 3 h into the scotophase. For storage, the extract was transferred to a small glass vial and diluted to obtain a concentration equivalent to the production of one female (FE) per 10 µL. GC analysis showed that 1 FE corresponded to approximately 20 ng of (*Z*,*E*)-9,11-tetradecadienyl acetate.

### Electrophysiological Experiments

Male moths were anesthetized with CO_2_ and restrained in a Styrofoam holder. A chloride-coated silver wire was inserted into the abdomen to serve as a reference electrode. One antenna was fixed with small strips of adhesive tape on the surface of the holder. Single sensillum recordings were obtained from trichoid hairs using the tip recording [27]. The tips of a few olfactory hairs were cut off using sharpened forceps. These sensilla were sampled among the long trichoid hairs that were previously shown to house one ORN tuned to (*Z*,*E*)-9,11-tetradecadienyl acetate [28], [29]. The recording electrode filled with sensillum saline (10^–3^ M Ca^++^ solution, according to the protocol by Pézier [30], which was modified from Kaissling and Thorson [31]) was slipped over the end of the cut trichoid hair. Both electrodes were connected to a NL 102 preamplifier (Digitimer, England). The signal was amplified (x1000) and band-pass filtered (0.2–10 kHz). It was digitized at 10 kHz and 12 bits with a Data Translation DT3001 board (Data Translation, Marlboro, USA). Spike firing was analyzed using Awave software [32] to detect and sort spikes and calculate the time of occurrence of individual spikes. Some recordings showed the spiking activity of two cells, which differed by spike amplitude. Only the firing activity of the spikes with large amplitude was attributed to a Ph-ORN and considered for analysis because the firing frequency of the other spikes was not modified by (*Z*,*E*)-9,11-tetradecadienyl acetate. Spike times were exported and saved as xls files.

Experiments started less than 1 min after connecting the recording electrode to a sensillum, and the recording session lasted less than 10 min for one sensillum.

### Olfactory Stimulation

Olfactory stimuli were delivered with the same programmable olfactometer as described in Party et al. [18], and distinct sources for Phero and PVs were used. Charcoal-filtered air was re-humidified and divided in 8 equal flows (220±10 mL/min), each directed to a 3-way miniature valve. Activating the appropriate valve directed the flow to the glass vial containing the stimulus source. The connections were made using polytetrafluorethylene tubing (PTFE) (1.32 mm ID). For PVs, the vial contained 1 mL of the appropriate dilution in mineral oil. The pheromone, diluted in hexane, was deposited into a section of PTFE tubing (1.6 mm ID; L = 20 mm) that was connected to the input needle of the vial after evaporation of the solvent. One milliliter of pure mineral oil was used as neutral stimulation. Stimulus and clean air carrying tubes were assembled together in a metal tubing of 100-mm length. A 1-mL disposable plastic pipette cone was placed at the output of the metal tubing to constitute a mixing chamber for the air flows coming from the eight tubes. The output of the cone was focused on the antenna with a micromanipulator. The cone was changed every day.

### Stimulus Sequences

Programming of the electric valves was performed using an 8-channel Valve Bank (AutoMate Scientific, USA). The antenna was continuously bathed in pure air delivered through 2 identical channels (220 mL/min each). During stimulation periods, one pure air channel was replaced by either odorized air (Phero or PV) or air passing over pure mineral oil (neutral stimulation). A triggering signal was used to synchronize the acquisition of the electrophysiological signal with the stimulation program. Phero was used at a 1-µg dose. PVs were used at 0.1%, 1% and 10% v/v dilutions in mineral oil. These doses at the source provided concentrations in air estimated at 1.9 ppb for Phero and 20 ppb to 3 ppm for linalool [18].

#### Effect of a background on pheromone response

A series of 4 stimulations at 1-min intervals was presented to every sensillum, in a different order from one sensillum to another. Odorant or neutral stimulation was applied either as a short single presentation, designated as “puff” (0.5 s), or a prolonged stimulation designated as “background,” which started 1 s before the puff and stopped 1 s after the puff (2.5 s). The series included a neutral puff (mineral oil) in a neutral background, a puff of Phero in a PV background, a puff of Phero in a neutral background, and a neutral puff in a PV background. A 2.5-s exposure time to the background was chosen because previous work showed that a maximum effect on Phero response was reached when the ORNs were exposed to Lin before Phero presentation, and to be able to monitor the decay of the Phero response while ORNs were still in odorized background [18]. To test the effect of Lin on Phero-sensitivity, every sensillum was stimulated with increasing doses of Phero (1 ng to 50 µg) in a neutral or Lin background (1%) and the two dose response curves were calculated.

#### Detection of plant volatile compounds

Electroantennography (EAG) was used to determine if the PVs were detected by the male antennae. Every antenna was stimulated with 3 doses of a PV: 0.1%, 1% and 10% v/v dilutions in mineral oil (different insects for Iso, Hex:Ac and Lin). Odorant or neutral stimulation was applied as a “puff” of 0.5 s.

### Data Analyses

The spike firing activities were analyzed using custom-written R scripts (http://www.r-project.org/). The means ± standard errors of the mean numbers of spikes emitted during the application of the stimuli were calculated for each treatment within series and compared using Wilcoxon signed-rank tests for paired data.

The firing rate was calculated using the local slope of the cumulative function of spike times [33]. Calculation of the slope was performed over a moving spike window of n-2, n+2 spikes (total of 5 spikes). Thus, each spike was attributed a firing rate and its occurrence time. The maximum rate (max rate) and the time of this maximum (peak time) between series (means ± standard errors of the mean) were compared using Wilcoxon signed-rank tests for paired data using custom scripts developed in R.

The amplitude of the EAG was measured with Awave and the differences between the responses to mineral oil and PVs were compared using Wilcoxon signed-rank tests for paired data.

### Behavioral Experiments

A locomotion compensator (LC-300, SYNTECH, Hilversum, The Netherlands) was used to record the movements of male *S. littoralis* in response to olfactory stimulation. The locomotion compensator is made of a 30-cm diameter sphere, on which the male is placed on top. Insect movements at the top of the sphere are recorded by an infrared light-sensitive camera positioned overhead. The digitized images provide coordinates of the center of gravity of the animal. This information is used to compensate for the insect displacement in real time by rotating the sphere using two electrical motors placed orthogonally to keep the center of gravity of the insect on top of the sphere. A virtual insect path is therefore obtained from the sphere rotation and stored as incremental X and Y coordinates.

To prevent them from flying off the sphere, moths were anesthetized with CO_2_ soon after their emergence, and their wings were removed 24 hours before the test. As described in *Bombyx mori* [3] or *Manduca sexta* [2], wingless male *S. littoralis* respond reliably to the pheromone by walking [19], [20]. Experiments were performed during the activity period of *S. littoralis*, 2 to 4 h into the scotophase with red light, at 22–24°C.

### Olfactory Stimulation

Olfactory stimuli were delivered with a programmable olfactometer adapted from Party et al. [18]. Air from the building was charcoal-filtered and divided into two flows in a Y-connector (model P514, Upchurch Scientific, Oak Harbor, USA). One flow was humidified and connected to the main branch of the “stimulation tube” to serve as constant flow (9.6 L/min). The second flow was divided in four 700 mL/min flows using a manifold (model P-115, Upchurch Scientific, USA). Each of the four flows was connected to a miniature electro-valve (model LHDA1233115H, the Lee Company, Westbrook, USA) driven by a Valve-Bank programmer (AutoMate Scientific, Berkeley, USA). The output of each valve was connected with PTFE tubing (1.32 mm ID, 20 cm L) to a hypodermic needle (18G size) inserted through the septum of a 4-mL glass vial. Another needle was used for the vial output. The vial for PVs contained 1 ml of MO solution. As a pheromone source, 1 µL of Phero diluted in hexane (at 1 µg/µL) was deposited in a piece of PTFE tube (1.32 mm ID, 15 mm L). After hexane had evaporated, the tube was slipped over the input needle into the vial. The male was constantly bathed by the constant humidified air flow, and at stimulus presentation, the neutral stimulus was replaced by odorized air and introduced in the constant flow. This maintained a constant moist/dry air ratio. A TTL signal was used to synchronize the acquisition of the walking path with the stimulation program. The stimulus sources were renewed daily. Contaminated air was removed from the set-up by an exhaust fan.

### Stimulus Sequences

Recordings of the walking path started approximately 20 s after placing the insect on the locomotion compensator. Five series of experiments were performed in which we varied the pattern of odor application. Experiments 1 to 4 lasted for 120 s and experiment 5 for 210 s. A graphic summary of olfactory stimulations is presented in Figure S1.

#### Experiment 1: constant odor presentation

This series of experiments was aimed at establishing the behavioral response of insects to MO (neutral stimulus), Phero, Lin, Hex:Ac and Iso applied continuously for 120 s. A dose-response curve to Phero (0.001 to 10 µg) in the absence or presence of Lin was also established.

#### Experiment 2: addition of PV

Phero was presented during the whole experiment while a PV (Lin, Hex:Ac, and Iso diluted at 0.1%) was added from the 60th second onward.

#### Experiment 3: behavior at Phero onset

Phero was presented after 60 s while either a neutral or a Lin background was presented to the insect throughout the entire experiment (0 to 120 s).

#### Experiment 4: loss of odor cue

At 60 s, we terminated delivery of the odor that was presented during the first phase of the trial to evaluate the effect of a loss of one component of the odor cue. The protocols were: Phero in neutral background followed by loss of Phero, Phero and Lin followed by loss of Phero, and Phero and Lin followed by loss of Lin.

#### Experiment 5: plasticity

The experiment duration was extended to 210 s to test the effect of novelty and/or habituation. Two conditions were tested: (i) three 30-s applications of Phero interrupted by 30-s stops of Phero in a neutral background; (ii) three successive 30-s Lin additions separated by phases of 30 s of clean air under constant Phero stimulation.

### Data Analyses

Walking paths were sampled at 10 data points/s and saved as csv files using TrackSphere (Syntech, Hilversum, The Netherlands). Data from the csv files were resampled (1 data point/s) and filtered before analysis using TRACKS, a program that was home-developed in R. The following parameters were calculated:

Activation = percentage of mobile insects. A male was scored as active when it walked continuously for a distance at least 3 times that of the body length (75 mm). This distance was calculated by adding contiguous individual steps of more than 0.5 mm to eliminate non-locomotory movements.
Orientation = percentage of active males whose tracks had a mean angle between -30 and +30° (0.52 rad) relative to the source.
Distance = median of the total walked distances for active (oriented or not) males in mm, presented with the median absolute deviation (mad).
Speed = median speed (mm/s) calculated during the periods of active locomotion of the active males.
Orientation index (oi) at t_i_ = cos (θ) * ρ, where θ is the angle and ρ the length of the mean vector at t_i._ of the tracks of the active moths [34].
Straightness (str) = ratio of the length of the final vector to the total distance walked, comprised between 0 and 1 (rectilinear path).

Pearson’s chi-squared tests were used to compare proportions of active and oriented males. To measure the effects of a stimulus, or of a change in background, we calculated the values for these parameters during time windows of the whole track, defined relative to the critical periods of the experiments (typically over 10-s windows before and after the stimulus changes during experiments 2–4). Values for the time windows sampled from the same track were compared using Wilcoxon paired tests and Fligner-Killeen tests of homogeneity of variance. Values for time windows taken from different treatments were compared using Mann-Whitney U tests. All statistical analyses were performed using custom-made R scripts.

### Photo Ionization Detector (PID)

To trace the olfactory stimulus we used a miniature photo ionization detector (PID) (Aurora Scientific Inc., Aurora, Canada). The ionization potential of (*Z*,*E*)-9,11-tetradecadienyl acetate lies above the energy of the lamp (10.6 eV), so that this compound cannot be detected by the PID, but Lin at 10% dilution was well detected. Thus, the variations in the concentration of the odorant in air were monitored using Lin to check the stability of the source and measure the dynamic of the stimulation. We applied 10 stimulations of 2 minutes of Lin with the same sources to test the stability and the selling out of the source. The PID probe was placed at the output of the delivering tube of the sphere. Signals from the PID were digitized and stored in a microcomputer using a DT9816 converting board (Data Translation) piloted by routines home-developed with Measure Foundry (Data Translation). Recordings of the PID signals were analyzed with specific applications developed in R.

### Wind Tunnel

A wind tunnel was also used to compare flying behavior with walking responses to Phero on the locomotion compensator. *S. littoralis* activity (% of activation and orientation) was compared between the two bioassays. Wind tunnel experiments were performed as described in Barrozo et al. [35] with 2- to 5-day-old males exposed to 10 µg of Phero for 3 minutes.

## Results

### Electrophysiology

#### PV detection

EAG recordings showed that male antennae responded to Lin, Iso and Hex:Ac in a dose-dependent manner. At the dilution used for background in the electrophysiology and behavioral experiments (0.1%), the EAG amplitudes for Lin (1.23 mV) and for Hex:Ac (0.54 mV) were significantly higher than the responses to mineral oil (below 0.03 mV), (Wilcoxon signed rank test, *P* = 0.002, *N = *13 for Lin and *P* = 0.003, *N* = 17 for Hex:Ac). For Iso, the difference was not significant at 0.1% (0.56 mV, *P* = 0.224, *N = *13), most likely due to a high level of responses to control (0.44 mV) compared to the other two series, but it was significant at 1% (1.27 mV, *P* = 0.002, *N* = 13).

#### Background

**Figure 1:**
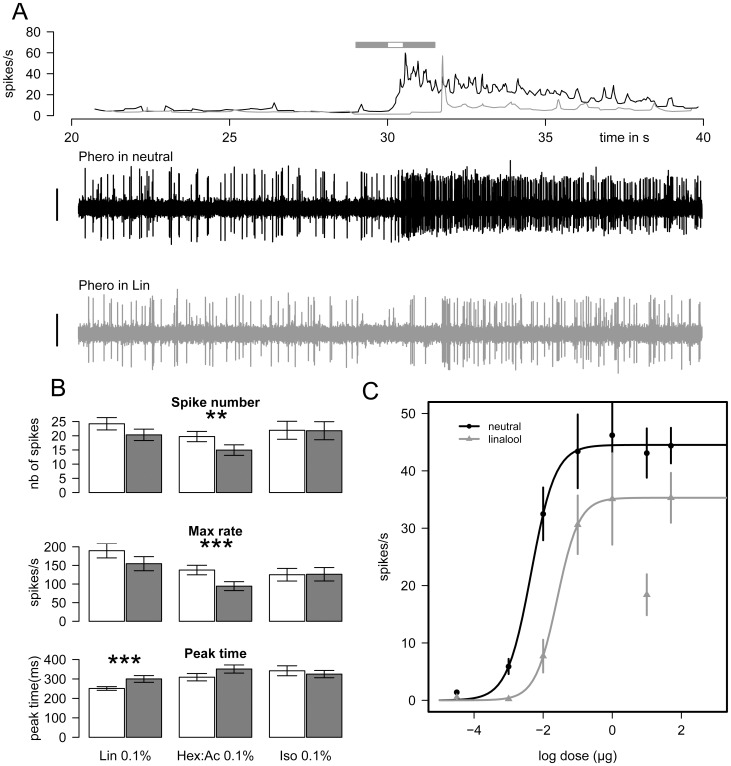
Lin and Hex:Ac backgrounds modify Ph-ORNs responses. A) Two examples of extracellular recordings from a Ph-ORN that showed a response to 1 µg Phero in neutral (middle trace, black) or Lin backgrounds (lower trace, grey) with corresponding frequency plots (upper graph; times of odor presentations are symbolized by a grey horizontal bar for the Lin background, and a white bar for Phero stimulus). Vertical bar = 1 mV. B) Responses of Ph-ORNs to Phero in the presence of neutral (white bars) or in PV backgrounds (grey bars, Lin, Hex:Ac or Iso at 0.1%). Bar plots present the number of spikes emitted during the pheromone stimulation (upper), the maximum firing rate (middle) and the peak time of the response (lower). Responses of Ph-ORNs in the different PV backgrounds were recorded from different insects (mean ± SEM, N = 20−22). Asterisks indicate significant differences at 1% for ** and 0.1% for ***, Wilcoxon signed-rank test for paired values. C) Dose-response curve to Phero in neutral (black curve) or Lin (grey curve) backgrounds (mean ± SEM, N = 13−30).

The intensity of the responses of Ph-ORNs to Phero in the presence of a PV background varied according to the PV. Firing responses in Lin and in Hex:Ac backgrounds were always lower compared to the response in the neutral background recorded from the same Ph-ORN, whereas Iso had no effect on the pheromone response (Figure 1A and B).

In presence of 0.1% Lin, the number of spikes emitted during the Phero stimulation was reduced (Wilcoxon signed rank test, *P = *0.052). The maximum firing rate was lower, but the difference was not significant (*P = *0.14). In turn, the peak time of the response was significantly delayed compared to the neutral background (*P = *0.0001). Between Phero stimulations, Ph-ORNs emitted 1.3±0.7 spikes in Lin 0.1% *vs.* 3.1±0.8 in a neutral background. This difference was not significant (Wilcoxon signed rank test, P = 0.1).

The background of 0.1% Hex:Ac significantly reduced the number of spikes fired by Ph-ORNs in response to Phero (*P = *0.008) and the maximum rate (*P = *0.0001). As with Lin, an upward trend was observed for the mean peak time of the response, but the difference was not significant (*P = *0.071).

The Iso background had no effect on the response of Ph-ORNs to Phero. Neither the number of spikes (*P = *0.98), the maximum firing rate (*P = *0.86), nor the latency of the response (*P = *0.64) were affected by it.

#### Dose response curve to Phero in neutral or Lin background

To test the hypothesis of a competition between pheromone and linalool molecules for binding on the active site of the pheromone, we compared dose response curves to Phero in a neutral versus Lin background (Figure 1C). In the neutral background, we obtained a sigmoid-shaped dose response curve with a plateau at 1 µg. With the Lin background, the dose response curve was shifted to the right and downward. At each individual dose, the responses to Phero were lower in the Lin background than in the neutral background. A strong dose of Phero did not prevent Lin inhibition, indicating non-competitive inhibition.

### Behavior

#### PID

The concentration of Lin at the output of the delivering tube of the locomotion compensator was stable during the two minutes of stimulation, as shown by the constancy of the PID signal. After 2 minutes it decreased by only 5.3% (mean of n = 4 different sources). When we applied ten 2-min stimulations with the same Lin source, the PID response was 30% stronger for the first stimulation than for the others, but the 9 following stimulations produced the same response. To homogenize stimulation intensity in behavioral experiments, we eliminated this first stronger release by delivering an initial stimulation before introducing the insect.

#### Response to natural gland extract or main pheromone compound

We first checked the level of the behavioral activity triggered by Z9E11-14:Ac stimulation in our locomotion compensator. We compared the PWR to the main compound of the pheromone blend (Phero, 1 µg) with the PWR to the female extract (1 FE). Natural extract and Phero elicited the same percentages of activation (70% and 85%, respectively, χ^2^ = 0.574, *df* = 1, and *P = *0.224) and orientation (55% and 65%, respectively, χ^2^ = 0.104, *df* = 1, and *P = *0.373). Phero at 1 µg thus reliably reproduced the activity of natural pheromone.

#### Wind tunnel vs. locomotion compensator

We compared walking responses of wingless males to Phero in the locomotion compensator to flight responses of intact males in the wind tunnel. A dose of 10 µg of Phero was used in the wind tunnel to take into account the dilution of the stimulus in a larger air volume in the tunnel. In the wind tunnel, we recorded 45.7% of activation and 34.3% of oriented flight, with the two scores being significantly lower than the proportions of activation (85%) and oriented walk (65%) in the locomotion compensator to 1 µg of Phero (χ^2^ = 5.69, *df* = 1, and *P = *0.009 and χ^2^ = 3.68, *df* = 1, and *P = *0.02, respectively).

These two experiments showed that male *S. littoralis* with wings removed readily respond by walking to the main component of the pheromone blend. Higher scores of locomotor activity were reached in the locomotion compensator compared to the wind tunnel. Further experiments were conducted in the locomotion compensator with wingless males and using a dose of 1 µg of Phero.

#### Experiment 1: “constant odor presentation”

**Figure 2:**
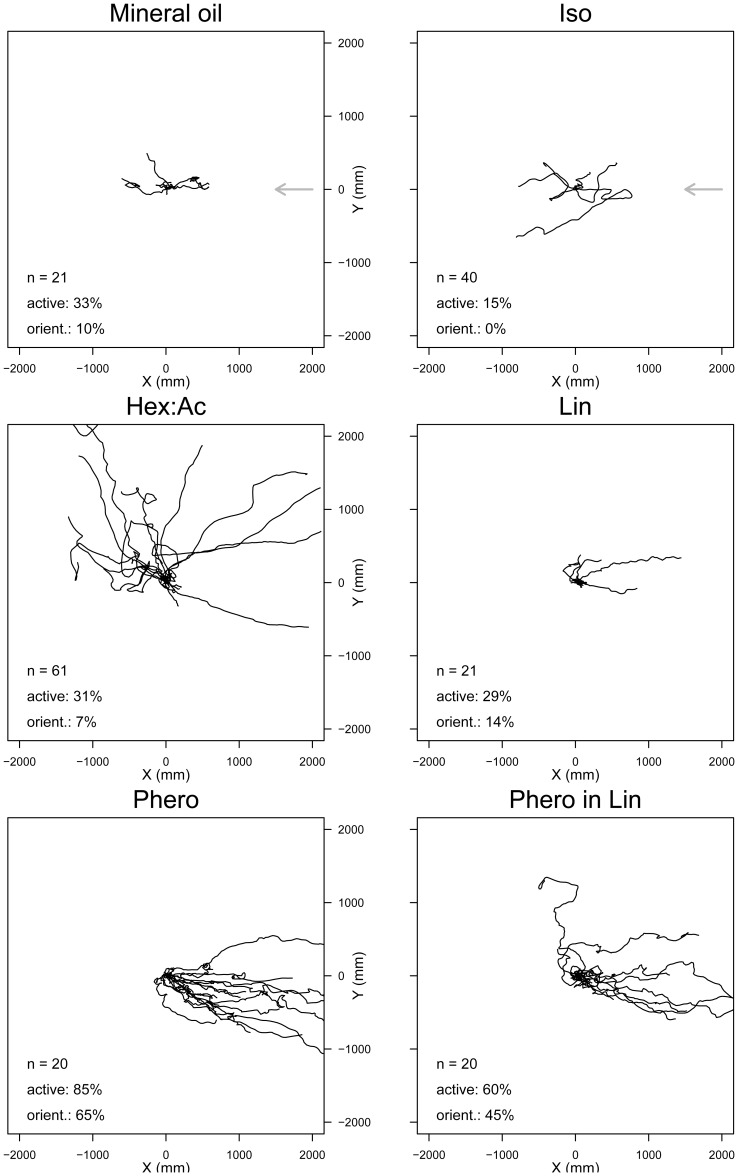
Pheromone but not plant odor stimulation triggers an oriented walking response in male *S. littoralis*. Surimposed walking tracks of individual males during constant stimulation with mineral oil, Iso, Hex:Ac, Phero, and Phero in Lin background for 2 minutes. Air was flowing from right to left (grey arrow in the 2 upper diagrams). X- and Y-axes indicate the cumulative walked distance in mm, relative to the release point at x0, y0.

The walking paths of male *S. littoralis* recorded while they were stimulated with different odorants for two minutes varied according to the stimulus (Figure 2).

With a neutral stimulation (mineral oil), only 33% of the males were activated and 10% oriented. Total walked distances (further reported as the median and (mad)) by active males, oriented or not, were short (median 569 (mad 524) mm). Similarly, the levels of activity and orientation to Lin and Iso were low: the median distance walked was ca. 900 mm for less than 30% active and less than 15% oriented males (Figure 2; median (mad): Lin: 874 (195) mm; Iso: 899 (735) mm). With Hex:Ac, the activation was the same as with Lin (31%), but walked distances were longer than with Lin (2217 (1101) mm, *P* = 0.013). Males generally walked randomly relative to the air flow, and only 7% of them oriented.

The proportions of active and oriented insects in response to Phero were the highest observed with respect to any other stimulation (85% of insects were active and 65% oriented to the source) (χ^2^ = 9.24, *df* = 1, and *P = *0.001 in comparison with neutral). Total walked distances were also longer than with Lin (1810 (997) mm, *P* = 0.003) (Figure 2). Males active during the 1^st^ minute maintained their locomotion during the 2^nd^ minute of the test.

When 0.1% Lin was applied simultaneously with Phero, the activity (60%) and orientation levels (45%) remained high and were not significantly different from Phero alone (χ^2^ = 2.01, *df* = 1 and *P* = 0.078 for activation and χ^2^ = 0.40, *df* = 1 and *P* = 0.26 for orientation). The total walked distance to Phero plus Lin was similar to Phero alone (2353 (952) mm, *P* = 0.33).

**Figure 3:**
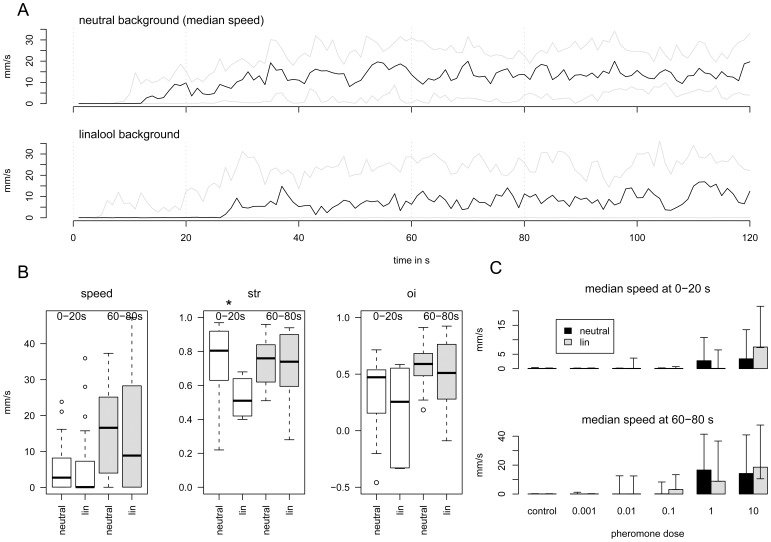
Analysis of the PWR in neutral or linalool background. A) Time plot of the median speed during the 2-min test in neutral or Lin backgrounds (median in black; first and third quartiles in grey, *N* = 20 for each group). B) Box plots of the median speed, straightness (str) and orientation index (oi) calculated within two 20-s time windows (delimited by vertical grey dashed lines in A) at the beginning (white bars) and the middle of the response (grey bars). In box plots, the bold line presents the median value, the limits of the boxes (hinges) are the first and third quartiles, the whiskers present the extremes and the dots the outliers. The values were compared with a Mann-Whitney test. Significant differences at 5% are indicated by *. C) Phero-dose response curves (median speed), measured within two time windows at the beginning and the middle of the recording for neutral (black bars) or Lin backgrounds (grey bars) (*N* = 20 for each group).

The evolutions of the median speed of the male sample in response to Phero in a neutral or in a Lin background are compared in Figure 3A. We analyzed the effects of the Lin background for speed, straightness, and orientation index measured at the beginning (0–20 sec, white bars) and in the middle of the experiment (60–80 sec, grey bars). Lower values were observed for the three parameters in both time windows analyzed in the Lin background compared to the neutral background. The difference was significant for straightness, which was 0.80 in the neutral background and 0.51 in the Lin background (Mann-Whitney test, *P* = 0.039).

No walking response was observed at lower doses of Phero (0.001 to 0.1 µg) in either neutral or Lin backgrounds. With the highest dose of Phero (10 µg), the median speeds in neutral and Lin backgrounds were not significantly different in either the first 20 s or in the middle of the experiment (60–80 s) (*P*>0.30; Figure 3C). Interestingly, during the first 20 s, in the Lin background, the median speed in response to 10 µg of Phero was significantly higher than the response to 1 µg, whereas in the neutral background, the plateau of the dose response curve was more rapidly reached: the response to 10 µg was the same as with 1 µg.

#### Experiment 2: “addition of PV”

**Figure 4:**
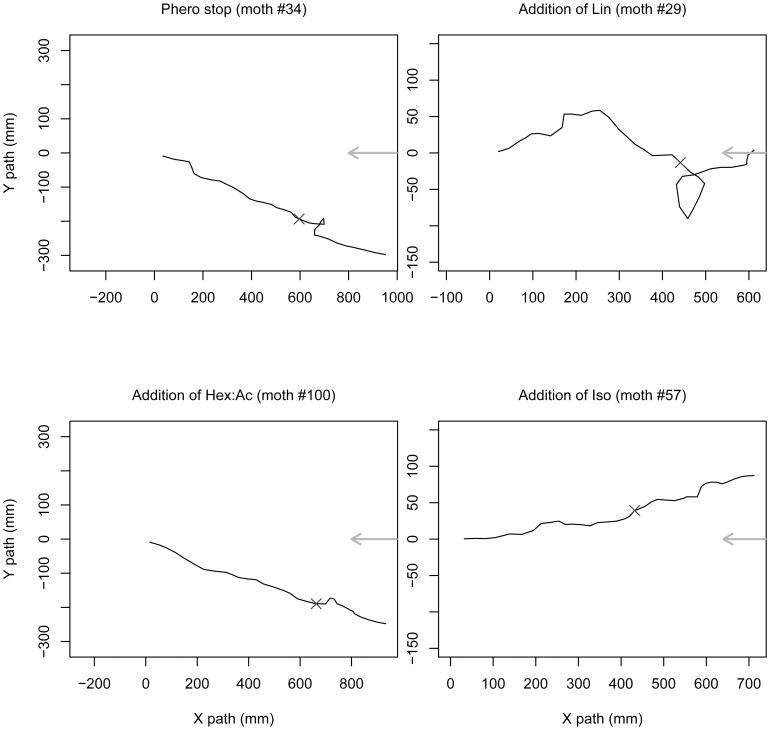
Changes in background modify the PWR. Examples of individual tracks that show the effects of changes in the odor background. Only the section of the whole track corresponding to the 40–80 s time window is presented. The background was changed at 60 s (cross). X- and Y-axes indicate the distance in mm. The direction of the air flow is indicated by the grey arrow.

The effects of the addition of a PV background while males were engaged in PWR were different according to the PV. As shown by examples of individual tracks, PWR were affected by the addition of Lin and Hex:Ac but not of Iso (Figure 4). Globally, males decreased their speed just after the Lin or Hex:Ac onset, walked in a loop for ten seconds, after which they resumed orientation towards the odor source (Figure 4 and S2). Their walking activity at PV onset was very similar to that observed at Phero offset (Figure 4, upper left).

**Figure 5:**
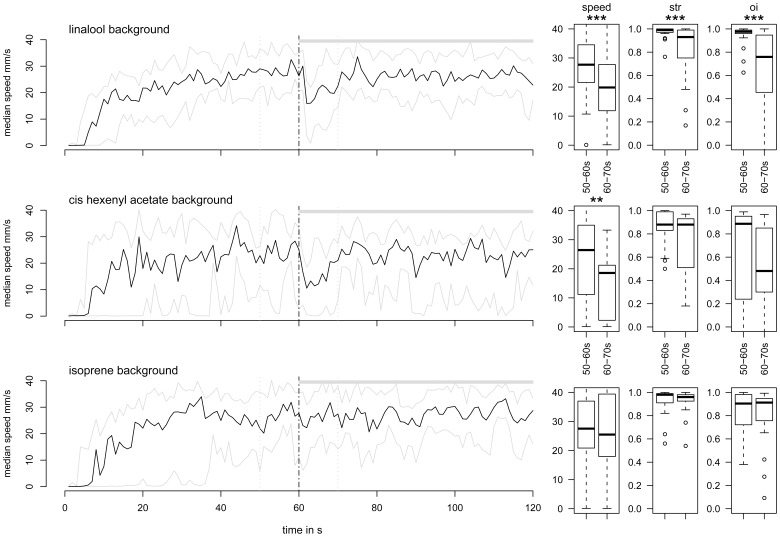
Addition of Lin and Hex:Ac to pheromone decreased transiently the walking speed. On the left: time plots of speed changes (median in black; first and third quartiles in grey). Upper horizontal grey bars show Lin (*N* = 27), Hex:Ac (*N* = 17) or Iso (*N* = 26) background presentation. On the right: box plots of the median speed, straightness (str) and orientation index (oi) calculated within two 10-s time windows (delimited by vertical grey dashed lines on curves at left), before and after the addition of PV. Box plot legends as in Figure 3. The values were compared with Wilcoxon paired tests. Significant differences are indicated by * for 5%, ** for 1% and *** for 0.1%.

To better characterize the effect of the change of background on the PWR, we compared speed, straightness and orientation index, measured before (50 to 60 s) and immediately after (60 to 70 s) the addition of the PV. Only the tracks of males that had presented an orientation response to Phero during the first phase were considered (Figure 5).

When the background of Lin was added to Phero, the speed, straightness and orientation index significantly decreased immediately (Wilcoxon signed rank test, *P*<0.001 in all cases). The median speed dropped from 27.7 to 19.9 mm/s. Similarly, the median of the orientation index decreased from 0.98 to 0.76 and the median of the straightness decreased from 0.99 to 0.93, while its interquartile range strongly increased at the Lin onset. Twelve males out of 27 showed a decrease greater than 5% in the straightness at the onset of Lin, while the fifteen other males were not affected. These decreases were temporary and short; the three parameters were no more significantly different in the 70–80 s time window compared to their value before the onset of background (*P* = 0.78, 0.44 and 0.15, respectively).

With the Hex:Ac background, we observed a significant decrease in the speed, from 26.4 mm/s before the change to 18.6 mm/s after the change (*P* = 0.008). Neither the median value for the orientation index (*P* = 0.56) nor its interquartile range (χ^2^ = 0.16 and *P* = 0.69) significantly changed. The median straightness was not affected by the addition of Hex background and was maintained at 0.88 (*P* = 0.29). Contrary to the Lin background, which decreased the speed and disturbed the orientation, Hex:Ac mainly affected the speed (Figure 5). We can exclude that the observed changes were due to artifact responses to the switching from one channel to another because when we switched between two channels, delivering Phero at time = 60 sec, males did not change their speed, straightness or orientation index (*P = *0.50, 0.82 and 0.22, respectively (data not shown).

Addition of Iso did not modify the PWR. None of the three parameters was affected by the Iso application (*P*>0.30).

#### Experiment 3: “behavior at phero onset”

**Figure 6:**
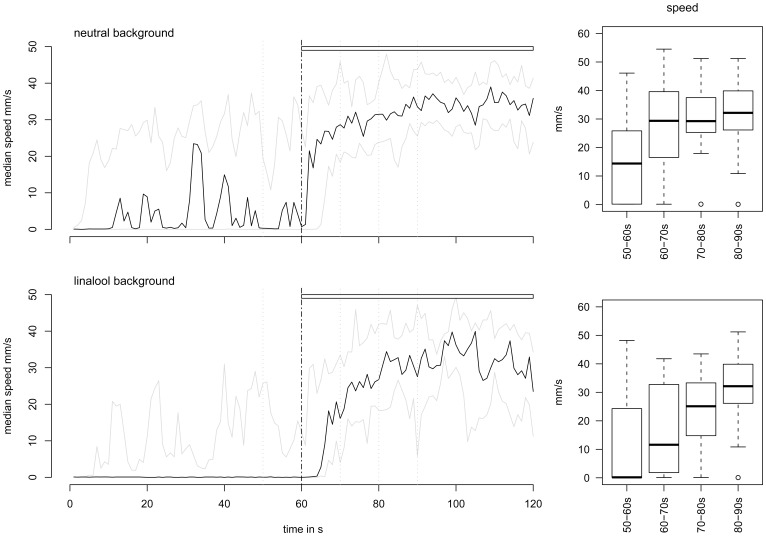
Lin delays the onset of the PWR. On the left: time plots of speed changes during two-phase experiments (median in black; first and third quartiles in grey). Upper horizontal bars show pheromone presentations. The vertical grey dashed lines delimit the four 10-s time windows chosen to calculate and compare the median speeds presented in the corresponding box plots (Right) prior to and after pheromone application in a neutral (*N* = 22) *vs.* a Lin background (*N* = 18). Box plot legends as in Figure 3.

We started the Phero stimulation at 60 s, either in a neutral or a Lin background, to measure the effects of background on the onset of male locomotion. As shown in Figure 6, the increase of walking speed after the onset of pheromone stimulation was slower in a Lin than in a neutral background. At Phero onset (60–70 s), the speed reached 29.4 mm/s in the neutral background, but only 11.6 mm/s in the Lin background (*P* = 0.07). The orientation index was also significantly greater in the neutral than in the Lin background (0.89 *vs.* 0.38, *P = *0.033). The straightness was not significantly different (0.98 in neutral and 0.92 in Lin backgrounds, *P* = 0.075). As in the previous experiment, the Lin effect was transient, and during the two following time windows (70–80 s and 80–90 s), the parameter values were similar between the neutral and Lin backgrounds (*P*>0.23).

#### Experiment 4: “loss of odor cue”

**Figure 7:**
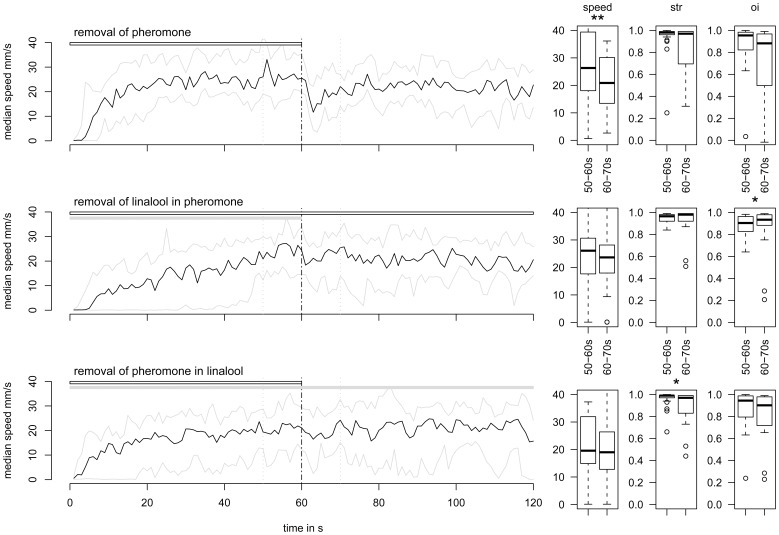
Lin could act as secondary cue to maintain the orientation after loss of odor. On the left: time plots of the evolution of speed during two-phase experiments (median in black; first and third quartiles in grey). The horizontal bars show PV (grey) and pheromone (white) presentations. The vertical grey dashed lines delimit the 10-s time windows before and after the loss of Phero in a neutral background (*N* = 24), the loss of Phero in a Lin background (*N* = 26) or the loss of Lin during Phero presentation (*N* = 24), and chosen to calculate and compare the median speeds, straightness (str) and orientation index (oi) presented in the corresponding box plots (Right). Box plot legends as in Figure 3.

At Phero offset, the median speed decreased from 26.3 to 20.9 mm/s (*P* = 0.008). Some males looped but no extended zigzag movements were observed. The median values of orientation index and straightness were not significantly affected (*P* = 0.46 and 0.61, respectively), while the interquartile ranges increased significantly for the straightness (χ^2^ = 5.51, *P* = 0.02), but not for the orientation index (χ^2^ = 0.62, *P* = 0.43). After 20 seconds, the orientation resumed (Figures 4 and 7).

At Lin offset in a background of Phero, speed and straightness were not significantly altered (*P* = 0.18 and 0.38, respectively). The orientation index increased slightly (from 0.90 at 50–60 s to 0.93 at 60–70 s, *P* = 0.038). Except for this slight increase of the orientation index, there was no global enhancement of the PWR, contrary to our expectations.

Stopping Phero in a background of Lin slightly but significantly reduced median straightness (from 0.99 to 0.97, *P* = 0.016) and significantly broadened the interquartile range (χ^2^ = 8.04, *P* = 0.005). Observing single males confirmed that straightness was differently affected according to the individual. It was reduced by >5% in 11 out of 24 males and by >45% in 4 of them, while it did not change in the other 9 males. Contrary to the removal of Phero in a neutral background, the median speed and orientation index were not affected (*P*>0.23).

#### Experiment 5: “Plasticity”

We extended the test period from 120 to 210 s to measure the effects of a repetition of stimulus changes on PWR.

**Figure 8:**
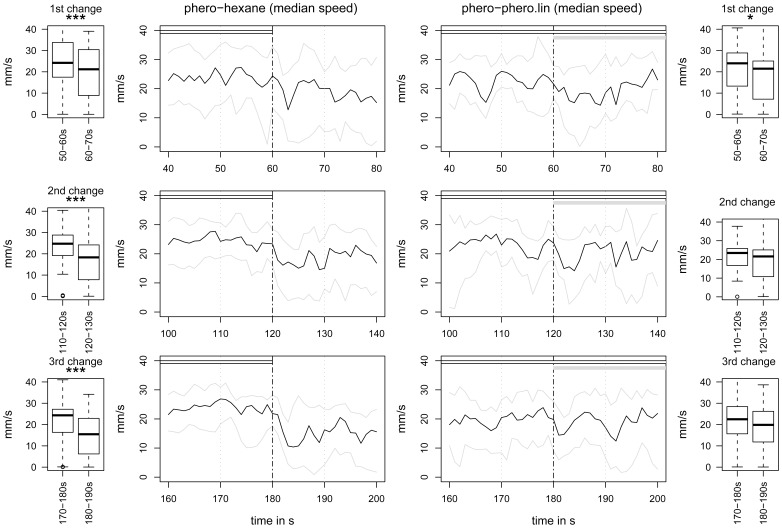
Males seem to habituate to successive Lin additions. Time plots showing the evolution of speed for three successive Phero stops (on the left, *N* = 36) and three successive additions of Lin (on the right part, *N = *22) and box plots showing the quantitative analysis of the speed median values during two 10-s time windows before and after each change (median in black; first and third quartiles in grey). The horizontal bars show PV (grey) and Phero (white) presentations. We calculated the speed values, presented in box plots upon two time periods (vertical grey dashed lines). Box plot legends as in Figure 3. The two time windows were compared with the Wilcoxon paired test. Asterisks indicate significant differences at 5% for * and 0.1% for ***.

When we stopped Phero 3 times for 30 s, all Phero stops evoked a significant decrease of speed (*P*<0.001) (Figure 8). The behavior was impacted, but for each change, the activity recovered after 10 s, returning to the same level as before the stop.

When we added Lin as a background to Phero 3 times for 30 s, we observed a significant decrease of median speed within the 10 s after the first Lin addition (*P* = 0.044) but not at the second and third additions (*P*>0.24 in all cases). The orientation index and the straightness were not significantly affected by Lin onset.

## Discussion

The analysis of the walking tracks of male *S. littoralis* clearly shows that PV application as a background to Phero affected the males’ PWR, Lin and Hex:Ac backgrounds, disturbing male responses to Phero. In turn, Iso did not affect PRW. The three compounds were well detected by the male *S. littoralis* olfactory system as indicated by the significant global EAG responses. Furthermore, the presence of ORNs responding to Lin and Hex:Ac in the male antennae was confirmed using single sensillum recordings from short hairs [36] (and A. Lemaire pers. comm.). Very few males walked actively in the presence of Lin or Iso alone. They were walking without preferred directions in the presence of Hex:Ac, showing neither clear attraction nor avoidance. Thus, the negative impact of Lin and Hex:Ac on PRW cannot be explained by repellency.

Our masking hypothesis involves the negative effect of the background on antennal Phero detection. We examined the effects of the three PVs on pheromone detection by specialist Ph-ORNs. As shown previously by Party et al. [18], the main effect of Lin on pheromone detection was a reversible reduction of the firing response to the main pheromone component. In addition, the present study shows that Lin application also affected the dynamic of the response: the firing activity reached its peak value later in presence of Lin than in a neutral background. Hex:Ac as a background decreased the number of emitted spikes and the maximum firing rate, but contrary to Lin, it did not affect the dynamic of the response. In turn, Iso background had no effect on the Ph-ORN response to Phero. Thus, the effects of the PVs on pheromone responses were compound-specific.

In insects, most studied examples of odor interactions are mixtures of host odors and anthropogenic repellents in mosquitoes. DEET, the most widely used topical insect repellent, blocks the behavioral attraction of female mosquitoes to lactic acid or other volatile host compounds. It was first proposed that blocking of the response of ORNs to host odor compounds by DEET is the main mechanism to explain how it inhibits the attraction to host odors [37]. However, later on, Syed and Leal showed that the reductions in electrophysiological responses were mainly due to the experimental design, a reduced amount of stimulus being released when DEET and the attractive odorant were combined in the same stimulus delivering cartridge due to a fixative effect of the less volatile DEET [38]. Furthermore, the authors showed that DEET is detected by specific ORNs and is avoided by mosquitoes in a sugar-feeding assay, inducing repellency in the absence of lactic acid [38]. In our experimental set-up, background and Phero were released from physically separate sources so that the background could not alter the emission of the Phero. Interestingly, Iso, which did not impede pheromone detection, did not disturb the orientation of males in the two-phase experiments. The fact that the PV effects on Ph-ORNs match their behavioral effects supports our hypothesis of molecular masking at the peripheral level.

A sudden stop in pheromone stimulation on the locomotion compensator evoked significant but short changes in the PWRs. Males performed local searches while walking, which resulted in a transient decrease in their speed and orientation index. We cannot exclude the presence of remnant pheromone molecules in the stimulation tube, which could be sufficient to keep the insect locked to the air direction. Nevertheless, the concentration of pheromone was considerably decreased, so we expected a more conspicuous decrease of speed and straightness. Opposite to our expectations, once launched, the directionality of the walk recovered rapidly on the locomotion compensator in the absence of the Phero stimulus, whereas male moths instantaneously changed their flight direction in a wind tunnel when they lost the signal.

Addition of a novel odorant during PRW produced different effects according to the compound, consistent with its effect on the detection of Phero by the ORNs. The addition of Lin had effects similar to a complete turn-off of Phero. Hex:Ac addition reduced the speed while inducing a loss of orientation in only some of the tested individuals. In turn, Iso addition had no effect on male PRW.

Surprisingly, the turn-off of Phero was less disturbing for males in a Lin background than in a neutral background. In a neutral background, the male moths were suddenly deprived of all chemical information, whereas in Lin, the air flow still contained odor cues after the end of Phero stimulation. This could explain why the Lin background apparently reduced the impact of the loss of Phero. Although this hypothesis must be confirmed by further experiments, it suggests that males are able to use general odors as subsidiary cues to supplement the specific signal. Males simultaneously perceived Lin and Phero during the first phase of the experiment so that an associative learning could be involved in that process, with Phero being the unconditioned stimulus.

According to the negative effects of Lin on Phero detection by Ph-ORNs, we expected that its removal would result in an increase in the PRW due to the recovery of the full capacity to perceive Phero. However, orientation to Phero and speed were not enhanced after we stopped the Lin background. Possibly, the male moths were fully habituated to the odor background so that their responses reached the same level as in a neutral background. Comparison of the PRW in neutral *vs.* Lin backgrounds reveals that after only 20 seconds, the PWR reached the same levels in both backgrounds, exhibiting the capacity of males to quickly adjust their behavior to the background.

Interestingly, the PRW was less affected by the Hex:Ac background than by Lin, although Hex:Ac decreased the firing response of Ph-ORNs more than Lin. The finding that Hex:Ac stimulated locomotion while males remained motionless in the presence of Lin might account for that discrepancy.

During its orientation flight toward a female in its natural habitat, a male moth encounters a variable combination of different plants so that its olfactory environment is constantly changing. It was thus important to examine its behavioral responses to repeated changes in background. Three consecutive interruptions of Phero had the same impact on PRW: after each stop, the male moth immediately decreased its walking speed and straightness, but PRW recovered rapidly to its former level. However, three consecutive additions of Lin resulted in a different pattern. Only the first addition significantly affected orientation, whereas no statistically significant effect was observed during the two following Lin additions. This attenuation of the effects of Lin on behavior during repetitive stimulation contrasts with its effect on antennal detection. Ten successive pulses of Lin produced the same decrease in the firing response of Ph-ORNs to Phero [18]. This suggests that the characteristic of novelty is also important in explaining the effect of a change in the odor background in addition to molecular masking.

The addition of Lin during the PRW was more disturbing for male walking behavior than the presence of a constant Lin background, suggesting that the change in the odorant environment induced an attention deficit in the insects, with the Lin surge acting as a “distracting stimulus”. Distraction is a phenomenon that is well documented in humans and other vertebrates [21], [23], [39], and it has also been observed in invertebrates. It can affect the performance of behaviors that are highly predictable and essential to survival, such as escaping from a predator. For instance, crabs take significantly more time to hide in the presence of predators when a noise is present, compared to no-noise experiments [22], [40]. Neil and Ellwood reported that an aquatic hermit crab can be distracted from ongoing activity by extraneous visual stimuli [41]. In locusts, Moorhouse et al. found that ambulatory behavior is interrupted by the presence of extraneous noise [42], [43]. Moth pheromone communication is also affected by external stimuli, such as bat ultrasounds [44], [45]. Interestingly, the effects of bat ultrasound exposure on the pheromone response are dependent on the quality of the pheromone signal. Most males flying towards high-quality pheromone sources quickly relocated the pheromone plume after ultrasound exposure and reached the odor source at a similar level as control males. However, those flying towards low-quality blends or non-optimal doses showed stronger reactions to the sound, and fewer reached the pheromone source. Thus, a male moth flying towards an odor stimulus of high quality seems ready to take greater risks than those flying towards odor sources of low quality. Our result reveals that besides acoustic or visual signals, olfactory stimuli can also be distracting stimuli to insects engaged in a motor task.

In conclusion, our results confirm the importance of a background of plant volatiles for pheromone communication in moths. They also confirm that general odorants can affect attraction to a specific olfactory signal by jamming its detection. However, the full range of consequences of a change in the olfactory background on behavior cannot be explained only by a molecular masking effect. This finding will contribute to a better understanding of the mode of action of deterrent or repellent chemicals, especially in strategies aiming to inhibit the response to a strong and specific attractant.

## Supporting Information

Figure S1 Olfactory stimulation protocols for the behavioral experiments. The horizontal bars show stimulations with PV (grey) and Phero (white) for the five series of experiments. Between odor stimulations, an equivalent flow of humidified air (dashed bars) ensured a constant rate of the total airflow.
(TIF)

Figure S2 Lin addition modifies the PWR. Video reconstruction of an individual track showing the effects of Lin addition on the PWR. When stimulated by Phero, a male *S. littoralis* walked upwind (white section of the track from 0 to 60 s). At addition of Lin (green track section, from 60 to 120 s), the male decreased its speed and walked in a loop for few seconds, after which it resumed its upwind orientation. The direction of the air flow is indicated by the arrow.
(MP4)
