# Supplementary figures and images for: Changes in Odor Background Affect the Locomotory Response to Pheromone in Moths
Source: PLoS One. 2013 Jan 2;8(1):e52897. doi: 10.1371/journal.pone.0052897 (PMC3534683; doi:10.1371/journal.pone.0052897)

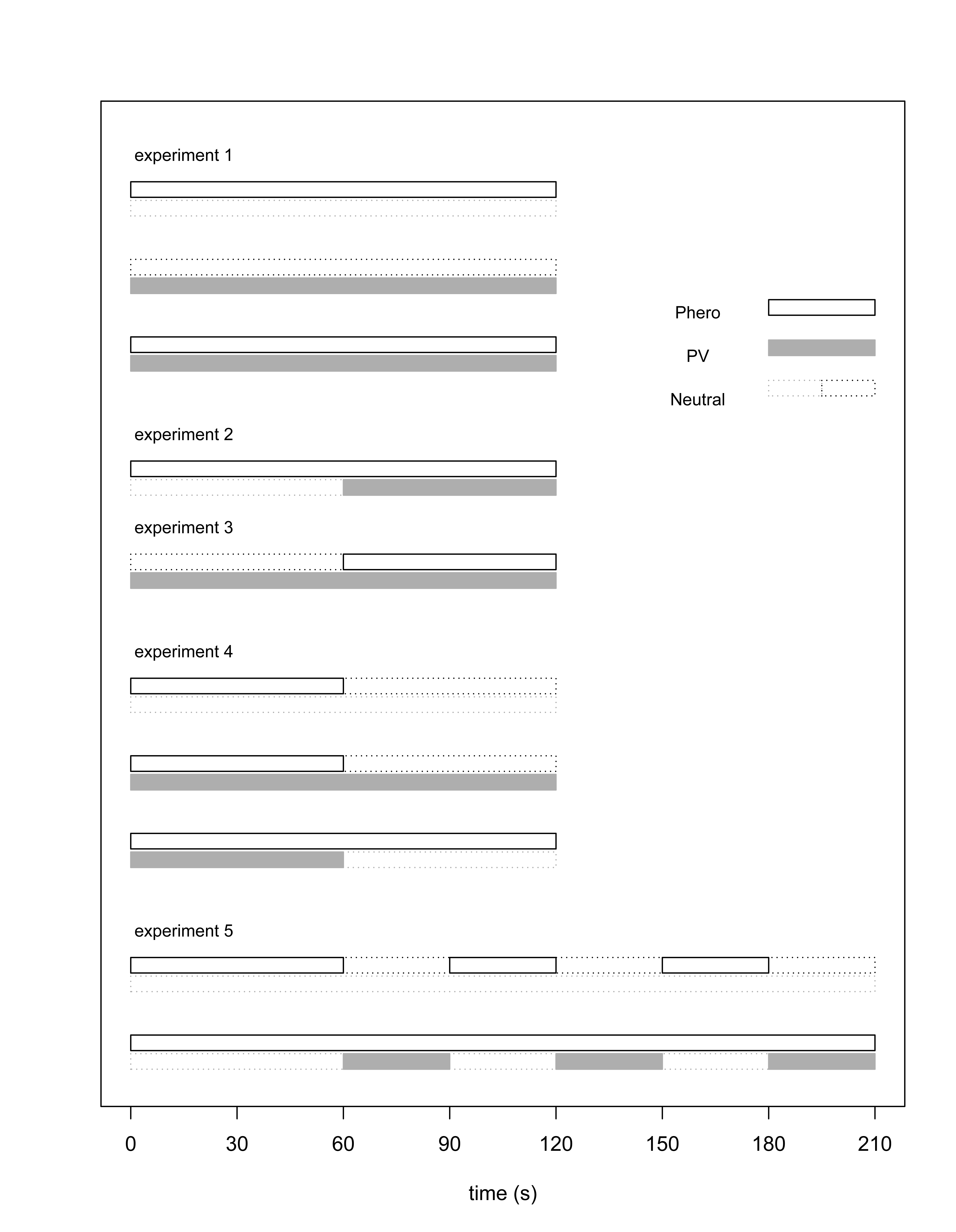

Supplement: Figure S1 — Olfactory stimulation protocols for the behavioral experiments. The horizontal bars show stimulations with PV (grey) and Phero (white) for the five series of experiments. Between odor stimulations, an equivalent flow of humidified air (dashed bars) ensured a constant rate of the total airflow. (TIF) [file pone.0052897.s001.tif]
